# Supplementary material for: Reproductive, maternal, newborn, child and adolescent health services in humanitarian and fragile settings: A mixed methods study of midwives’ and women’s experiences
Source: PLOS Glob Public Health. 2024 Jul 3;4(7):e0003384. doi: 10.1371/journal.pgph.0003384 (PMC11221643; doi:10.1371/journal.pgph.0003384)
Supplement: Attachment — Submitted filename: Peer Review 17th December 2023 PLOS One.pdf [file pgph.0003384.s001.pdf]

Peer Review 17th December 2023 PLOS One Dr Maeve O'Connell Assistant Professor Midwifery

Thank you for the opportunity to review this high quality and significant novel study which will advance the profession of midwifery. I consider this article to be worthy of publication. It is well written and well considered overall. The study is well crafted and appears to comply with the ethical concerns of compiling a complex and comprehensive international study. The rationale for the study and study objectives were clear. The results are well-presented and the implications of the study are teased out.

I really enjoyed reading this paper and could not find any issues with it. Perhaps the discussion could incorporate more comparisons with previous literature, however I believe this study is novel and so there are probably limitations in terms of this. I am satisfied with this. Therefore, I recommend its publications without any further edits.
