# Supplementary material for: Reproductive, maternal, newborn, child and adolescent health services in humanitarian and fragile settings: A mixed methods study of midwives’ and women’s experiences
Source: PLOS Glob Public Health. 2024 Jul 3;4(7):e0003384. doi: 10.1371/journal.pgph.0003384 (PMC11221643; doi:10.1371/journal.pgph.0003384)
Supplement: Attachment — Submitted filename: Reviewer 1 Revision 1 Comments.pdf [file pgph.0003384.s003.pdf]

Cancel Save and Close

PGPH-D-23-01467R1
"Reproductive, maternal, newborn, child and adolescent health services in humanitarian and fragile settings: a mixed methods study of midwives' and women's experiences"
Revision 1

Paulomi Niles (Reviewer 1)

**Reviewer Recommendation Term:**	Accept
**Rate Review:**	0
**Custom Review Question(s):**	**Response**
**Comments to the Author**<br><br>1. If the authors have adequately addressed your comments raised in a previous round of review and you feel that this manuscript is now acceptable for publication, you may indicate that here to bypass the "Comments to the Author" section, enter your conflict of interest statement in the "Confidential to Editor" section, and submit your "Accept" recommendation.	All comments have been addressed
2. Does this manuscript meet PLOS Global Public Health's publication criteria? Is the manuscript technically sound, and do the data support the conclusions? The manuscript must describe methodologically and ethically rigorous research with conclusions that are appropriately drawn based on the data presented.	Yes
3. Has the statistical analysis been performed appropriately and rigorously?	I don't know
4. Have the authors made all data underlying the findings in their manuscript fully available (please refer to the Data Availability Statement at the start of the manuscript PDF file)?<br><br>The PLOS Data policy requires authors to make all data underlying the findings described in their manuscript fully available without restriction, with rare exception. The data should be provided as part of the manuscript or its supporting information, or deposited to a public repository. For example, in addition to summary statistics, the data points behind means, medians and variance measures should be available. If there are restrictions on publicly sharing data—e.g. participant privacy or use of data from a third party—those must be specified.	Yes
5. Is the manuscript presented in an intelligible fashion and written in standard English?<br><br>PLOS Global Public Health does not copyedit accepted manuscripts, so the language in submitted articles must be clear, correct, and unambiguous. Any	Yes

typographical or grammatical errors should be corrected at revision, so please note any specific errors here.	
6. Review Comments to the Author<br><br>Please use the space provided to explain your answers to the questions above. You may also include additional comments for the author, including concerns about dual publication, research ethics, or publication ethics. (Please upload your review as an attachment if it exceeds 20,000 characters)	comments addressed.
7. PLOS authors have the option to publish the peer review history of their article (what does this mean?). If published, this will include your full peer review and any attached files.<br><br>**Do you want your identity to be public for this peer review?** If you choose "no", your identity will remain anonymous but your review may still be made public.<br><br>For information about this choice, including consent withdrawal, please see our Privacy Policy.	**Yes:** P. Mimi Niles
**Confidential to Editor**<br><br>1. Do you have any potential or perceived competing interests that may influence your review? Please review our Competing Interests policy and declare any potential interests. If you have no competing interests, please write "I have no competing interests."	n/a
2. Did you receive any assistance in preparing this review (e.g. from a post-doc or graduate student)? If yes, please include their name below. (optional)	no
3. If accepted, do you think this submission should be highlighted on the journal website and/or to the media? (optional)	Yes, on the journal website only
**Do you want to get recognition for this review on a Web of Science researcher profile?**<br><br>If you opt in, your Web of Science profile will automatically be updated to show a verified record of this review in full compliance with the journal's review policy. If you don't have a Web of Science profile, you will be prompted to create a free account.	Yes

More Reviewer Details

Cancel Save and Close
