# Supplementary material for: Reproductive, maternal, newborn, child and adolescent health services in humanitarian and fragile settings: A mixed methods study of midwives’ and women’s experiences
Source: PLOS Glob Public Health. 2024 Jul 3;4(7):e0003384. doi: 10.1371/journal.pgph.0003384 (PMC11221643; doi:10.1371/journal.pgph.0003384)
Supplement: Attachment — Submitted filename: Rebuttal Letter v0.1 21DEC.docx [file pgph.0003384.s002.docx]

Rebuttal letter

We thank the reviewers and editors hugely for the time taken to review the article. The feedback and guidance has been most helpful. Please do find below a summary of our responses.

**Journal Requirements**

**1. Please review your reference list to ensure that it is complete and correct. If you have cited papers that have been retracted, please include the rationale for doing so in the manuscript text, or remove these references and replace them with relevant current references. Any changes to the reference list should be mentioned in the rebuttal letter that accompanies your revised manuscript. If you need to cite a retracted article, indicate the article’s retracted status in the References list and also include a citation and full reference for the retraction notice.**

We have reviewed carefully and also included the additional reference suggested by Reviewer 1.

**2. Please provide separate figure files in .tif or .eps format only and remove any figures embedded in your manuscript file. Please also ensure all files are under our size limit of 10MB.**

This has been done

**3. Figs 1 & 3: please (a) provide a direct link to the base layer of the map (i.e., the country or region border shape) and ensure this is also included in the figure legend; and (b) provide a link to the terms of use / license information for the base layer image or shapefile. We cannot publish proprietary or copyrighted maps (e.g. Google Maps, Mapquest) and the terms of use for your map base layer must be compatible with our CC-BY 4.0 license.**

This has been done. Please also note the following:

One additional layer was utilized for the [ocean bottom from Natural Earth](https://www.naturalearthdata.com/downloads/50m-raster-data/50m-ocean-bottom/). This is open data in the public domain (PDcc). A citation for Natural Earth data is not necessary, but if a citation is desired there is [information here](https://www.naturalearthdata.com/about/terms-of-use/).

To comply with WHO legal policy, include the following disclaimer language in any publication these maps appear:

*The designations employed and the presentation of the material in this publication do not imply the expression of any opinion whatsoever on the part of WHO concerning the legal status of any country, territory, city or area or of its authorities, or concerning the delimitation of its frontiers or boundaries. Dotted and dashed lines on maps represent approximate border lines for which there may not yet be full agreement.*

**Additional editor comments**

There were no additional editor comments highlighted to address.

**Reviewers**

*Reviewer 1*

**Authors provide a timely and valuable review of experiences of midwives and service users in HFS settings.**

**I am honored to review this manuscript. It is well written and conceptualized. I write this review as a practicing midwife- working in a low resource, urban setting in the US and a researcher of midwifery care services and workforce issues that impact midwives caring for systematically and historically marginalized peoples.**

Thank you so much for your review and your helpful feedback.

**Is there a empirical definition of HFS? Please provide. What constitutes HFS?**

There is no consensus on a single definition for Humanitarian and Fragile Settings and it is a broad term. Often, the states do overlap. To try and ensure a working definition we created a composite index categorising countries across a spectrum of fragility. This was based on the triangulation of data from five classifications of humanitarian and fragile states, that is, Organisation for Economic Co-operation and Development (OECD), the World Bank, the Fragile State Index, the INFORM Severity Index, and the United Nations Office for the Coordination of Humanitarian Affairs (UNOCHA) data on humanitarian response plans. We have detailed this within the methods section of the paper.

**Pg 2: authors state “Considering the close link they have to the communities, midwives are also uniquely placed to provide a full range of SRMNCAH people-centred care services beyond immediate maternal and newborn care.12 This is especially important in HFS where health systems are broken or fragile and access is inconsistent. It is estimated that midwives can provide 90% of essential SRMNCAH services and as such are critical for optimum, cost-effective service delivery.13 In addition to preventing maternal and newborn deaths, quality midwifery care can also improve over 50 other health-related outcomes, including - sexual and reproductive health, immunisation, breastfeeding, tobacco cessation in pregnancy, prevention and management of communicable and non-communicable disease and obesity in pregnancy, early childhood development, and postpartum depression.14 Since they work across all levels of care, from communities to hospitals, midwives are uniquely placed to provide essential services to women and newborns in the most difficult settings.1”**

**While all of this promotes the values of midwifery care - my concern is that this expectation of this type of value driven care is not fully actionable in the face of acute structural and resource instability in HFS. The way it is stated may misconstrue this possibility. I am weary of communicating that midwifery can offer this level of service coverage in ALL contexts, midwifery can offer this value in its ideal form. Can authors make sure this is restated in this paragraph? I too often see midwifery care being promoted as a singular solution to poor outcomes when in fact we know that using the QMNC framework requires that the entire ecosystem be invested in. Suggest “WHILE…. Midwives can provide 90% of SRMH…. We caution that these may be severely restricted in HFS” – otherwise you reify the false notion that midwives can make something out of nothing.**

We very much agree with the reviewer of the need for concerted and comprehensive investment across the health system. And also take on the point of nuancing the capacity of midwives when in the context of a supportive and enabling environment to do so. We have rephrased as such.

Study Design

**Authors could explicitly state which elements are quant and which elements are qual - this is not clear anywhere. “A concurrent mixed methods approach was applied, using secondary analysis of primary quantitative and qualitative data sources.” Do authors have any other studies or methods papers that use this type of methodology to cite? It is uncommon.**

Thank you- weve added a citation to Cresswell 2003 to provide detail on this particular mixed methods approach.

**Please name the four countries first and then explain how data was obtained or used.**

This is described within the Qualitative Data and Quantitative Data section in the methods.

**Convergence – can authors explain why Greece was not included in the quantitative synthesis?**

Greece itself was not an HFS state. However the qualitative data provided critical information from refugee camp settings which was important to add depth to the review.

**Appreciative of the qualitative descriptive synthesis. Authors should state that this is a descriptive qualitative synthesis - not interpretive, as these are two distinct fields in the methodological space.**

Thank you for flagging this- we have ensured to mention this.

**How are authors naming the participants in the quotations? What do these lettesr/numbers mean that are at the end of each quote? Describe to readers or simplify, type of participant and place - otherwise it is difficult to decipher.**

Thank you for this suggestion. As this is a secondary analysis, to ensure consistency across the countries we have simplified to type of participant and place.

**It is also not clear who these midwives are? Are they midwives from the countries that they are serving, are they foreign trained aid workers?**

The qualitative studies included in the analysis did not specify between these two categories and so it is unfortunately not possible for us to specify this.

**Of note - please do not use female and women interchangeably - female denotes biological category, woman denotes social/gendered category. I suspect authors mean women when they write female. Please correct throughout.**

Thank you for flagging this important point- we have ensured to correct throughout.

**The convergence of data is the weakest aspect of this analysis. In essence there is very little convergence as they are in separate categories and presented separately. In true convergence, authors make the connections between these two data types for readers clear and decipherable.**

To clarify, this is not a convergent approach but a concurrent or parallel approach. As such the quantitative and qualitative strands of the research are performed and analysed independently, and their results are brought together for interpretation. We have highlighted the overall interpretation within the discussion section as per convention.

**The discussion section is compelling. Would authors consider taking out the implications for practice and put those in a separate section? You have a future work section and It may be beneficial to take all the recommendations that are scattered throughout the discussion and make a bulleted list that would be in the implications/future directions section.**

Thank you for this helpful suggestion. We have done this and specified by Research, Policy and Clinical action.

*Reviewer 2*

**Congratulations to the team on this novel research! I consider this article to be worthy of publication. It is well written and well considered overall. The study is well crafted and appears to comply with the ethical concerns of compiling a complex and comprehensive international study. The rationale for the study and study objectives were clear. The results are well-presented and the implications of the study are teased out.**

Thank you so much for your review and kind feedback.
