# Supplementary material for: Reproductive, maternal, newborn, child and adolescent health services in humanitarian and fragile settings: A mixed methods study of midwives’ and women’s experiences
Source: PLOS Glob Public Health. 2024 Jul 3;4(7):e0003384. doi: 10.1371/journal.pgph.0003384 (PMC11221643; doi:10.1371/journal.pgph.0003384)
Supplement: Attachment — Submitted filename: Rebuttal letter.docx [file pgph.0003384.s004.docx]

Rebuttal letter

We thank the reviewers and editors hugely for the time taken to review the article. The feedback and guidance has been most helpful. Please do find below a summary of our responses.

**Journal Requirements:**

**We have noticed that you have uploaded Supporting Information files, but you have not included a list of legends. Please add a full list of legends for your Supporting Information files after the references list.**

The supporting information file was the rebuttal letter and response to editors- this is not supporting information to be added to the manuscript. Therefore this has not been added to the manuscript.

**Additional editor comments**

**Acronym DRC appears in Page 4 in the 5^th^ Paragraph: studies were in Afghanistan 34, the DRC 35, and South Sudan 36. Please give the full form of the acronym here**

**Full form of DRC - Democratic Republic of Congo Appears in Page 5. Data from 75 midwives were included; 72% of midwives were from the Democratic Republic of Congo (n=54), 4% of midwives from Afghanistan (n=3), 20% of midwives from refugee camps in Greece (n=15), 4% of midwives from South Sudan (n=3).**

We have corrected this.

**Reviewers**

All reviewer comments addressed. There were no additional reviewer comments highlighted to address.
